# Underwater Turbulence Detection Using Gated Wavefront Sensing Technique

**DOI:** 10.3390/s18030798

**Published:** 2018-02-14

**Authors:** Ying Bi, Xiping Xu, Sing Yee Chua, Eddy Mun Tik Chow, Xin Wang

**Affiliations:** 1 School of Engineering, Monash University Malaysia, Jalan Lagoon Selatan, Bandar Sunway 47500, Malaysia; 2015100214@mails.cust.edu.cn (Y.B.); eddy.chow@monash.edu (E.M.T.C.); 2 College of Optoelectronic Engineering, Changchun University of Science and Technology, Changchun 130022, China; xxp@cust.edu.cn; 3 Lee Kong Chian Faculty of Engineering and Science, Universiti Tunku Abdul Rahman, Bandar Sungai Long, Kajang 43000, Malaysia; sychua@utar.edu.my

**Keywords:** underwater turbulence, gated wavefront sensing, time-of-flight

## Abstract

Laser sensing has been applied in various underwater applications, ranging from underwater detection to laser underwater communications. However, there are several great challenges when profiling underwater turbulence effects. Underwater detection is greatly affected by the turbulence effect, where the acquired image suffers excessive noise, blurring, and deformation. In this paper, we propose a novel underwater turbulence detection method based on a gated wavefront sensing technique. First, we elaborate on the operating principle of gated wavefront sensing and wavefront reconstruction. We then setup an experimental system in order to validate the feasibility of our proposed method. The effect of underwater turbulence on detection is examined at different distances, and under different turbulence levels. The experimental results obtained from our gated wavefront sensing system indicate that underwater turbulence can be detected and analyzed. The proposed gated wavefront sensing system has the advantage of a simple structure and high detection efficiency for underwater environments.

## 1. Introduction

The underwater detection technique has been widely used in oceanic explorations, fisheries, and military applications [1]. In these detection techniques, turbulence is a common problem in various types of fluids. Underwater turbulence has a significant effect on the momentum, heat, and diffusion of matter in water [2,3]. In general, the effect of light beam transmission in water by 1 m is equivalent to the transmission of 800–1000 m in the air [4]. Water temperature, density, and water disturbance will influence the water refractive index during underwater light transmission. This causes a minimal angular scattering of light, wavefront distortion, and a reduction of system resolution [5,6].

A lot of research has been done on underwater turbulence detection techniques over the past few years. Levine et al. used an autonomous underwater vehicle (AUV) [7] to measure the turbulence using two piezoelectric shear probes, an FP07 thermistor, and three orthogonal accelerometers mounted on a sting at the forward end of the vehicle. Meanwhile, a type of airfoil shear probe has proven to be an effective tool for measuring underwater turbulence [8]. An underwater glider is an ideal carrying platform for the airfoil shear probe, and can provide a means for underwater turbulence measurement [9,10,11]. However, the process of underwater turbulence measurement using an underwater glider has many limitations. Due to the movement characteristics of the underwater glider, the range and accuracy of the turbulence measurement will be affected [12].

Nowadays, wavefront sensing has been applied to multiple applications, such as: applied optics [13,14], ophthalmology [15,16], and optical detection [17,18]. The Shack–Hartmann wavefront sensor (SHWS) is a critical optical instrument in wavefront sensing [19,20,21]. It provides a method of estimating the incident wavefront upon a larger aperture by dividing the larger aperture into a number of smaller subapertures. With the development of wavefront sensing, it has also been applied to the field of underwater turbulence detection. Restaino et al. [22] proposed a method to detect underwater turbulence using the wavefront sensing technique and adaptive optics [23,24,25]. In their study, they used heating and cooling plates that can create a well-controlled thermal gradient that in turn generates different degrees of turbulence. Due to the influence of the water refractive index, they use a deformable mirror (DM) to correct the effects of turbulence and improve the performance of the optical system. The control signal generated by the wavefront processor controls the movement of the DM to compensate for the wavefront distortion [26]. Since the corrections are performed in real time, the control system has very high requirements, and thus does not have applicability [27]. Goncharovm et al. [28] proposed a method to correct the atmospheric turbulence using a linear atmospheric dispersion corrector (ADC) coupled with a deformable mirror and wavefront sensor on a large telescope. Wu et al. [29] designed a plenoptic sensor to retrieve the phase and amplitude changes resulting from a laser beam’s propagation through atmospheric turbulence. The reconstructed phase is obtained by multiple wavefront extraction techniques at various depths in a channel.

Laser sensing is highly advantageous for machine vision due to its non-contact and non-destructive nature. Over the past decade, major advances have been made to benefit applications in surveillance, military, industry, etc. [30,31]. Range gated technique has been a promising laser ranging method applied in night-vision environments. This technique can eliminate the backscattering effect during the laser propagation in underwater conditions [32,33]. The laser range-gating technique utilizes pulsed lasers and gated cameras [34,35]. The scattered light at different distances and the target reflected light are separated by controlling the camera’s gating time in order to acquire the image [36,37]. Therefore, time-of-flight (TOF) [38,39] is a crucial foundation in the range-gated technique. The principle of TOF is based on the propagation time of the optical signal between the object and the sensor, and the propagation speed of the optical signal to determine the distance [40,41,42]. The underwater imaging system researched by Mclean et al. [43] is a typical application of the laser distance gating technique. The system can be mounted on unmanned underwater vehicles to work in the deep sea. It can achieve a real-time measurement in an underwater environment, and it doesn’t need a complex control system to correct the wavefront distortion.

In this study, we combine wavefront sensing and the time-of-flight (TOF) range-gated principle to detect underwater turbulence. The gated wavefront sensing technique provides a simple structure, high detection efficiency, and strong anti-interference ability [44,45,46]. The timeliness deficiency of wavefront sensing could be remedied by introducing the time-of-flight technique. Our setup simulates the underwater turbulence using different flow rates of water. We can elaborate the characteristics and principle of the range-gated imaging, which operates based on the TOF principle in later sections. By using the proposed gated wavefront sensing system, the turbulence effect on the underwater detection is analyzed, and the experimental results are discussed in this paper.

## 2. Turbulence Detection Using Gated Wavefront Sensing Technique

### 2.1. Gated Wavefront Sensing

The traditional wavefront sensing technique samples the incident wavefront by means of a lenslet array, which produces an array of spots on a detector, such as Charge Coupled Device (CCD) camera. Basically, wavefront sensing is a measurement method that compares the reference focus with the small disturbances of the focus centroid from the measured wavefront. The displacement of each centroid location enables the computation of the wavefront slopes. The target surface can be reconstructed using the slope matrix. The advantages of the wavefront sensing technique are the simplicity of configuration, real-time processing, and high dynamic range. As water temperature, density, and moisture disturbances can affect the refractive index of water, conventional wavefront sensing techniques cannot effectively detect underwater turbulence. The range-gated technique is a well-known method to enhance the image quality and eliminate the backscattering effects during the laser propagation in water. Therefore, we propose a new method by combining the wavefront sensing and the range-gating principle. 

**Figure 1:**
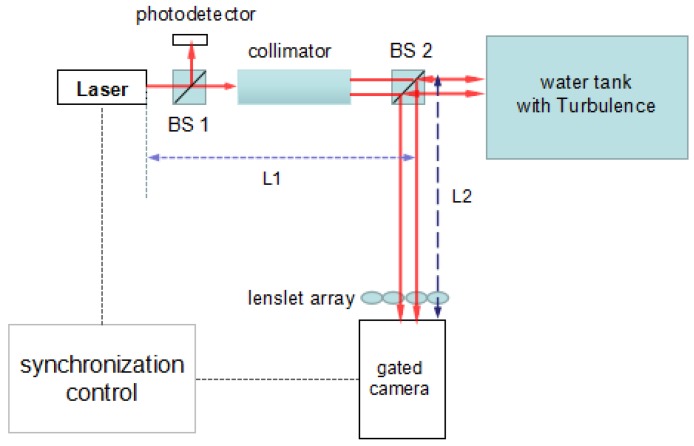
Schematic diagram of the gated wavefront sensing system.

We design a gated wavefront sensing system to detect the turbulence effect underwater, as illustrated in Figure 1. The system components are a pulsed laser, beam-splitting (BS) prisms, a collimator, a photodetector, the wavefront sensing assembly (e.g., lenslet and gated intensified CCD camera), and delay generator for system triggering and synchronization. The range-gating process starts when the laser emits a pulse towards the target in the water tank. As the light travels, it is absorbed and scattered. The camera gate is kept closed at all times, until the laser pulse returns to the camera after hitting the target. Once the reflected laser pulse is received, the camera gate is closed again to avoid any scattering effect interfering with the original image.

Light passes through a series of components that are separated by a distance along the light path, *r*. This can be expressed as follows according to the TOF principle:

(1) $$r=\frac{vt_{0}}{2},$$

where $t_{0}$ is the travel time of the laser pulse, and *r* is the speed of light in the working condition. In our setup, we set the distance from the laser to the second prism, BS2, as equal to the distance from the BS2 prism to the range-gated camera (L1 = L2), as shown in Figure 1, in order to simplify the calculation. The front part of each laser pulse will carry the wavefront information of the underwater turbulence from different distances.

### 2.2. Synchronization Control

**Figure 2:**
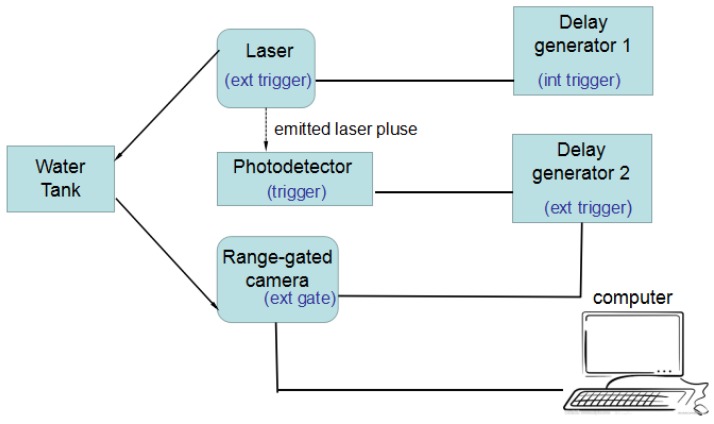
Synchronization control of the gated wavefront system.

Synchronization control between the pulsed laser and camera is particularly important in the proposed gated wavefront sensing system. Laser pulses are controlled in conjunction with the shutter speed of the camera to capture the returning pulse from the target, which contains the information required for wavefront reconstruction. Figure 2 illustrates the working principle of the synchronization control.

**Figure 3:**
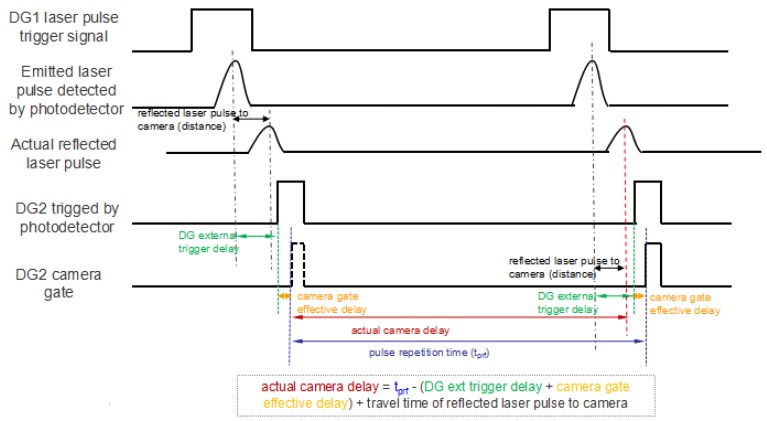
Timing diagram of the synchronization control.

As shown in Figure 2, there are two delay generators in the setup. The first delay generator is used to trigger laser pulses at a configured frequency. In order to have a better estimation on the arrival time of the laser pulses, a second delay generator is introduced. This delay generator is triggered by the photodetector when it detects the emitted laser pulse, and accordingly controls the range-gated shutter time. However, the delay generator has an unavoidable external trigger delay during the triggering process. This causes the first laser pulse to be missed when the second delay generator is triggered to open the camera gate. System calibration is necessary for an accurate determination of gate-opening time. This is one of the challenges in setting up an accurate system for obtaining a good quality turbulence reconstruction surface. The calibration of the system is performed in multiple stages. The calibration is split into two parts: the external trigger delay of the delay generator, and the effective delay of the camera. Figure 3 illustrates our design of synchronization control, and the timing diagram of the important signals in the experimental setup.

A range-gating process starts when the laser emits a pulse. The camera gate is kept closed at all times, and is only opened for a short time when the laser pulse returns to the camera after hitting the target. Thus, only light that arrives at the sensor within the right timing window can contribute to the imaging process. After synchronization, the gated wavefront sensor samples the incident wavefront from different interfaces by means of a lenslet array. The wavefront is spatially sampled and focused by the lenslet array on the camera.

### 2.3. Wavefront Reconstruction

In our gated wavefront sensing system, a lenslet array is used to focus the incoming wavefront onto a range-gated camera. This microlenslet array partitions the reflected wavfront into a larger number of smaller wavefronts, each of which is focused on a small spot on the sensor. The spatial displacement of each spot is a direct measure of the local slope of the incident wavefront as it passes through the lenslet. The integration of these slope measurements can reconstruct the shape of the wavefront.

**Figure 4:**
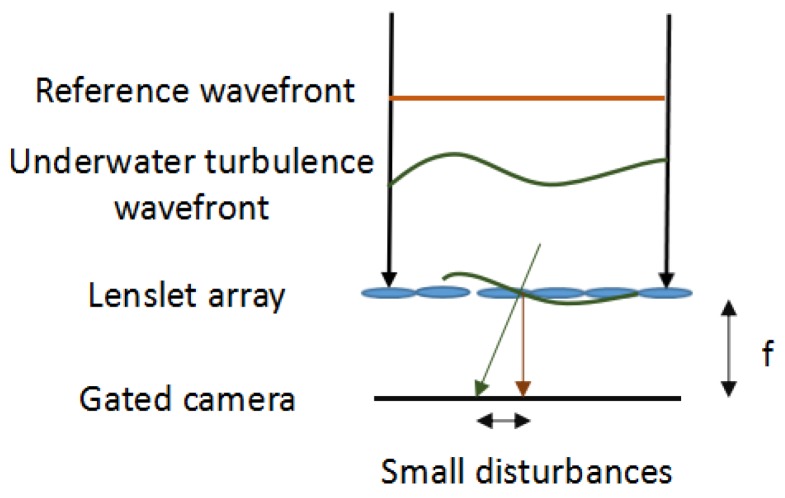
Schematic diagram of the wavefront sensor.

The centroid locations of the focal points are then compared with the reference focal points. The displacement of each centroid location reflects the wavefront slope. The wavefront information obtained at a water velocity of 0 L/min is used as the reference wavefront. The schematic diagram of the wavefront sensor is shown in Figure 4. 

Small disturbances, $\frac{\partial W(x,y)}{\partial x}$ and $\frac{\partial W(x,y)}{\partial y}$, are introduced to the sampling aperture. The centroid displacements reflect the wavefront slope change, $\frac{\partial W(x,y)}{\partial x}$ and $\frac{\partial W(x,y)}{\partial y}$, which are defined as:

(2) $$\frac{\partial W(x,y)}{\partial x}=\frac{\Delta x}{f},\frac{\partial W(x,y)}{\partial y}=\frac{\Delta y}{f}.$$

where $\Delta d_{x}$ is the average slope over a subaperture diameter in the x direction; $\Delta d_{y}$ is the average slope over a subaperture diameter in the *y* direction; Δx is the measured spot centroid displacement from the reference in the *x* direction; and *f* is the focal length of the lenslet array.

After the focal spots were detected, we used the threshold method to improve the spot centroid extraction accuracy. There are many factors that can affect the signal-to-noise ratio (SNR) of the time-gated camera, such as photo shot noise, dark current noise, and fixed pattern noise. In our situation, photo shot noise plays a major role in all kinds of noises. In order to improve the SNR, we apply the thresholding algorithm. This algorithm filters out low-frequency and high-frequency noise, and improves the accuracy of spot centroid extraction in the wavefront reconstruction. The grayscale value of the light intensity of the spot image is given a weightage in order to highlight the influence of the central light intensity. On the basis of roughly determining the position of the centroid of the light spot, a window where the light spot is located is determined according to the position of the obtained centroid. Accordingly, the center of each spot can be calculated as follows:

(3) $$X_{c}=\frac{\sum_{i,j}^{L,M}x_{i}I_{ij}^{\alpha }}{\sum_{i,j}^{L,M}I_{ij}^{\alpha }},$$

(4) $$Y_{c}=\frac{\sum_{i,j}^{L,M}y_{j}I_{ij}^{\alpha }}{\sum_{i,j}^{L,M}I_{ij}^{\alpha }}.$$

where $X_{c}$ and $Y_{c}$ are the centroid position of the spot; $I_{ij}^{\alpha }$ is the αth high intensity value in this spot sub-area; $x_{i}$ and $y_{j}$ are the coordinates of the pixel in the whole spot image; and *L* and *M* are the numbers of the pixels along *x* and *y* directions in the window, respectively.

The slope matrix, or curvature matrix, is obtained for further reconstruction using an iterative method in orderto generate the surface. In this study, we use the Zernike polynomials. A distorted wavefront *W*(*x*, *y*) is represented by a Zernike polynomial as:

(5) $$W(x,y)=\sum_{i=0}^{k}C_{k}Z_{k}(x,y).$$

The purpose of reconstructing the wavefront is to solve each Zernike coefficient, *C_k_*. The gated wavefront sensor measures the slopes of the wavefront at each subaperture *A*(*i*,*j*). The total of the measurement points is N=m×n, where *m* is the number of rows, and *n* is the number of columns. From the distortion wavefront expression in the *x*, *y* direction, the wavefront slope can be expressed as:

(6) $$\frac{\partial W(x,y)}{\partial x}=\sum_{i,j=0}^{k}C_{k}\frac{\partial Z_{k}(x,y)}{\partial x}=\frac{\Delta x}{f},$$

(7) $$\frac{\partial W(x,y)}{\partial y}=\sum_{i,j=0}^{k}C_{k}\frac{\partial Z_{k}(x,y)}{\partial y}=\frac{\Delta y}{f}.$$

It can be described by a matrix equation:

(8) S=ZC.

The above formula (8) is expanded to obtain the matrix form as follows:

(9) $$\left|\begin{array}{c} \begin{array}{c} \frac{\partial Z_{111}(x,y)}{\partial x},\\ \vdots \end{array}\begin{array}{c} \frac{\partial Z_{112}(x,y)}{\partial x}\\ \vdots \end{array}\begin{array}{c} ,\ldots , \end{array}\begin{array}{c} \frac{\partial Z_{11k}(x,y)}{\partial x}\\ \vdots \end{array}\\ \begin{array}{c} \frac{\partial Z_{mn1}(x,y)}{\partial x},\\ \vdots \end{array}\begin{array}{c} \frac{\partial Z_{mn2}(x,y)}{\partial x}\\ \vdots \end{array}\begin{array}{c} ,\ldots , \end{array}\begin{array}{c} \frac{\partial Z_{mnk}(x,y)}{\partial x}\\ \vdots \end{array}\\ \begin{array}{c} \frac{\partial Z_{111}(x,y)}{\partial y},\\ \vdots \end{array}\begin{array}{c} \frac{\partial Z_{112}(x,y)}{\partial y}\\ \vdots \end{array}\begin{array}{c} ,\ldots , \end{array}\begin{array}{c} \frac{\partial Z_{11k}(x,y)}{\partial y}\\ \vdots \end{array}\\ \frac{\partial Z_{mn1}(x,y)}{\partial y},\frac{\partial Z_{mn2}(x,y)}{\partial y},\ldots ,\frac{\partial Z_{mnk}(x,y)}{\partial y} \end{array}\right|\left[\begin{array}{c} C_{0}\\ C_{1}\\ \vdots \\ C_{k} \end{array}\right]=\left[\begin{array}{c} S_{x11}\\ S_{x22}\\ \vdots \\ S_{xmn}\\ S_{y11}\\ S_{y22}\\ \vdots \\ S_{ymn} \end{array}\right],$$

where $\frac{\partial Z_{mnk}(x,y)}{\partial x},\frac{\partial Z_{mnk}(x,y)}{\partial y}$ denote the partial derivatives of the *k*th Zernike polynomial in the *x* and *y* directions within the *m*th rows and *n*th columns’ subaperture, respectively. *C_k_* is the coefficient of the *k*th Zernike polynomial to be solved. *S_xmn_* and *S_ymn_* are the measured wavefront slopes in the *x* and *y* directions on the *m*th rows and *n*th columns’ subaperture, which is a known amount that can be obtained by measurement. The coefficient matrix *C* can be calculated using the least squares method as:

(10) $$C={\left(Z^{T}Z\right)}^{-1}Z^{T}S=Z^{+}S.$$

The wavefronts are fitted to Zernike polynomials, as they are a very good description of wavefront aberrations. The Zernike polynomials allow us to characterize the aberrations by decomposing them to their respective coefficients. 

The reconstructed wavefront is then calculated using Equation (5) and the coefficients found. The deconvolution of blurred images can then be done using the wavefront obtained. The acquired image, *v*, and its correlation with the original image, *u*, can be described as:

(11) v = H(u) + p.

where *p* is the noise from image acquisition and the inaccuracies in wavefront reconstruction, and *H* denotes the convolution operator resulting from the reconstructed wavefront. The deconvolution of the acquired image would then be a minimization function [47]. In order to obtain the original image, *u*, the following equation is used:

(12) $$u^{\prime }=\underset{u}{\min}\left\{\frac{1}{2}\|H\left(u\right)-{p\|}_{2}^{2}+\Delta \|u\|\right\}.$$

with ∆ being the regularization parameter. 

## 3. Experimental Results and Discussion

### 3.1. Experimental Setup

**Figure 5:**
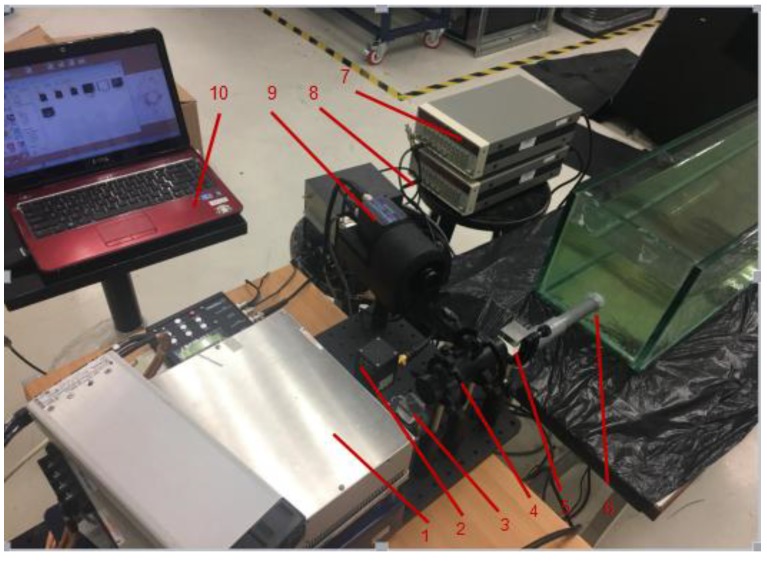
Experimental setup of the proposed gated wavefront sensing system. 1—laser; 2—photodetector; 3—BS1; 4—collimator; 5—BS2; 6—water tank with turbulence; 7—delay generator 1; 8—delay generator 2; 9—gated wavefront sensing assembly (lenslet and intensified CCD (ICCD) camera); 10—computer.

The experimental setup to detect underwater turbulence using the proposed gated wavefront sensing technique is shown in Figure 5. A WEDGE HB 532-nm wavelength was used as the laser source, and the 9450 series gated intensified CCD (ICCD) camera system, combined with a lenslet, were used as the gated wavefront sensing system. The water tank created turbulence at a distance of four meters from the laser source. The laser pulse passed through the collimation system, traveled toward the turbulence, and back to the gated wavefront sensing system, where the image was captured. As explained in Section 2.2, two delay generators were used in the synchronization control to the range gate at the distance of interest. Experiments were carried out in a long water tank. The turbulence level was simulated by controlling the flow rate of the water. Other environmental factors, such as the temperature of the water, were kept constant, as we only considered the effects of spatial turbulence due to the flow of water.

Actually, water disturbance, density, temperature, and other non-uniform factors will cause a random change in the refractive index of macromolecules in water. Underwater turbulence causes small changes of the refractive index (Δ*n* ≈ 10^−6^), which caused a very small angular scatter of the beam. In general, the bidirectional reflectance distribution function (BRDF) is an accurate description of the surface of the light reflection of the basic parameters. However, the underwater environment cannot be expressed by a simple negative exponential decay function. So, it is more difficult to measure its intensity distribution by conventional methods. The reflection ratio of light intensity in an underwater situation is about 20–30%. Therefore, we use a 532-nm laser and a time-gated camera to reduce the impact of underwater reflectance, and get the focal imaging.

**Figure 6:**
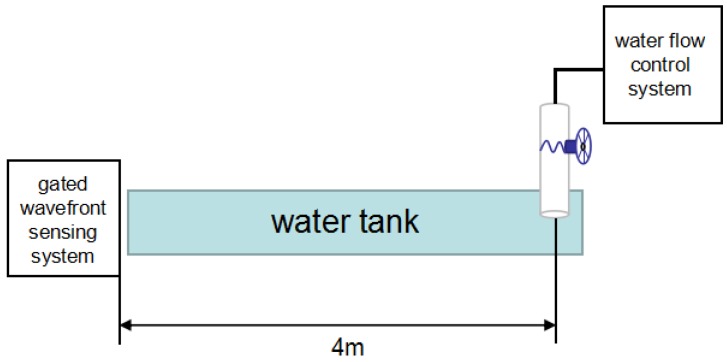
Gated wavefront sensing system and water tank control system.

In our synchronization control system, water flows into the tank at a distance of four meters from the wavefront sensing system. The flow rate can be controlled to create a turbulence condition. Figure 6 shows the gated wavefront sensing system and the water tank control system.

**Figure 7:**
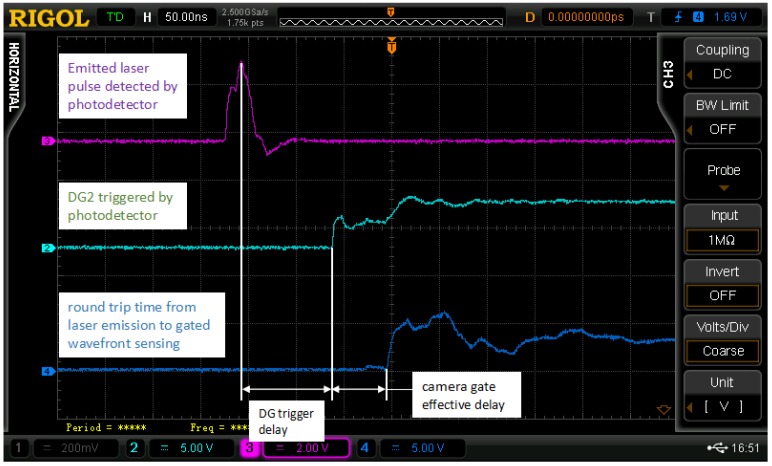
Determination of the actual delay generator (DG) trigger and camera delay time. The output from the photodetector shows in purple color; the output from the delay generator (DG) 2 shows in green color; and the camera gate output shows in blue color.

The actual delay time is calculated based on the principle of synchronization control, which was mentioned above. Figure 7 shows the three signals on an oscilloscope, where the time difference between each other i.e., the external trigger delay of the delay generator, and the effective delay of the camera, can be determined. The output from the photodetector is the emitted laser pulses (in purple); the output from the delay generator (DG) 2 is triggered by the photodetector (in green); and the camera gate output (in blue) is used as a reference to indicate the round trip time from laser emission to gated wavefront sensing. In our experiments, the laser pulse frequency was set to 30 Hz. To configure an accurate gate-opening time, we measured 50 times to calculate the standard deviation of the actual DG, and camera delay. From our calibration, a DG external trigger time delay is 116.2 ns, and the effective time gate delay is 11.4 ns.

Regarding the sensitivity and the dynamic range of the sensor, the gated wavefront sensor is limited by the focal length and the pitch of the microlens array, as well as the pixel size of the camera used. The microlens array we used in our setup has a focal length of 6.7 mm and a pitch of 150 µm, and the ICCD camera has a pixel size of 17.8 µm. Therefore, the corresponding sensitivity $\left(\theta_{\min}\right)$ and dynamic range $\left(\theta_{\max}\right)$ can be calculated accordingly. The calculation equations are as follows:

(13) $$\theta_{\min}=\frac{P}{f}=\frac{17.8\times 10^{-3}}{6.7}=0.002657\mathrm{rad}=0.1522{}^{\circ},$$

(14) $$\theta_{\max}=\frac{D}{2f}=\frac{150\times 10^{-3}}{2\times 6.7}=0.011194\mathrm{rad}=0.6414{}^{\circ}.$$

Subsequent experimental results show that the sensitivity and the dynamic range of the sensor can be effectively measured.

### 3.2. Results and Discussion

**Figure 8:**
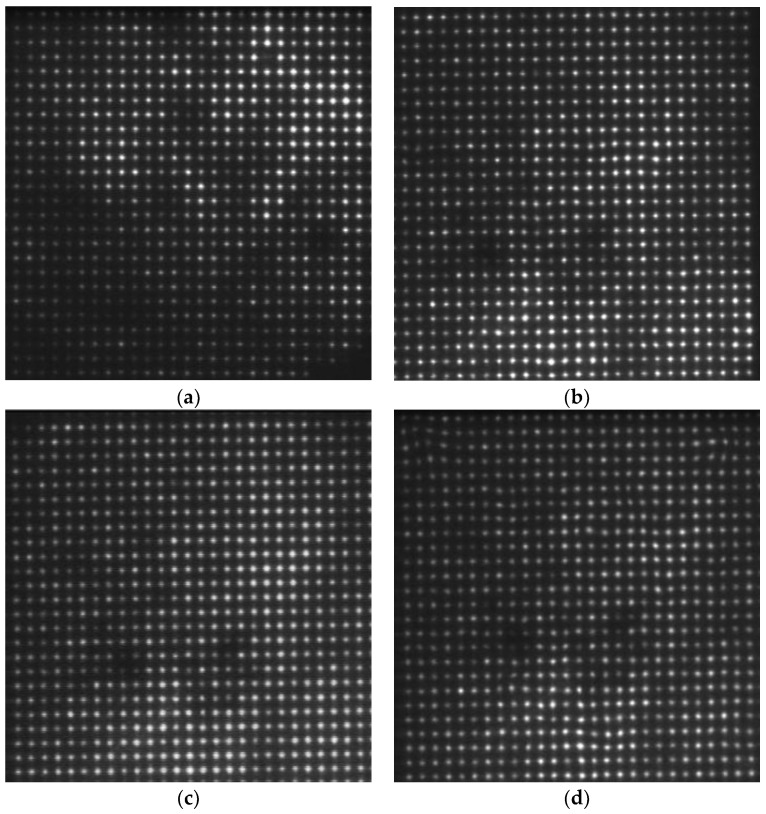
Focal spot images acquired from different distances. (**a**) Focal spot image from 1 m; (**b**) focal spot image from 2 m; (**c**) focal spot image from 3 m; (**d**) focal spot image from 4 m.

We acquired the focal image with the flow rate of 0 L/min as a reference for wavefront reconstruction. Four distances were selected for turbulence comparison, namely: 1 m, 2 m, 3 m, and 4 m. The water flow rate was set at 40 L/min. Figure 8 shows the focal spot images from different distances in the water tank with turbulence.

**Figure 9:**
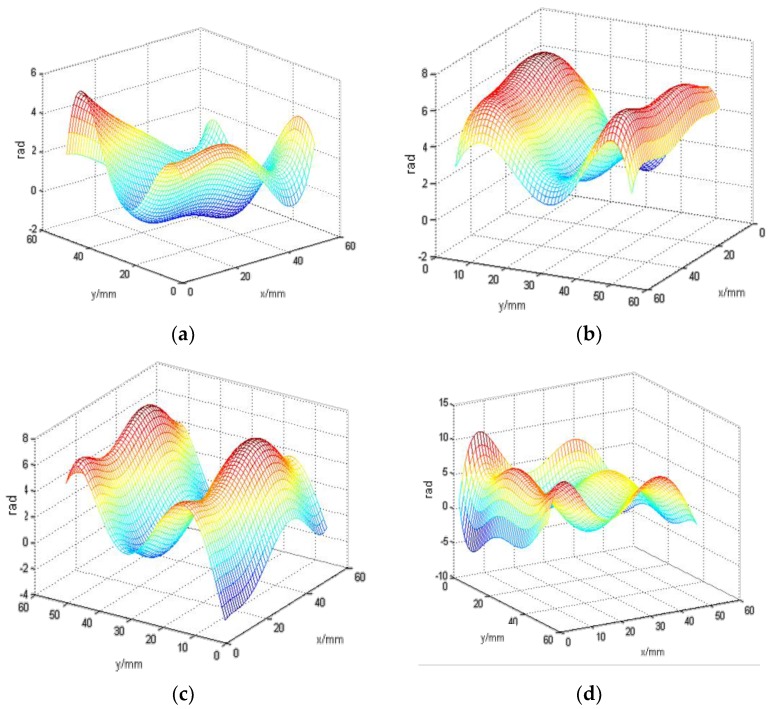
The results of wavefront reconstruction at different distances: (**a**) 1 m; (**b**) 2 m; (**c**) 3 m; and (**d**) 4 m.

The dark area in this focused image is a result of the light sensor receiving insufficient light intensity, which was caused by the camera’s shutter, the underwater scattering light conditions, or other unspecified factors. It will have a slight effect on the calculation of the center shifts. Therefore, we used the thresholding method in the centroid extraction to reduce this error and get a better reconstructed image. In order to observe the underwater turbulence effectively, we used the Zernike polynomials for wavefront reconstruction. In our underwater situation, the gradient reconstruction method is considered. Underwater turbulence is affected by many factors, such as water temperature, density, etc. We focused on one factor, which is the water flow rate. Due to the way that turbulence simulation is controlled by the water flow velocity changes in the water, we considered the gradient reconstruction method. This method has a faster processing speed. It can reconstruct objects more specifically. The offset was obtained by subtracting the center of reference focal spots from the measured focal spots and obtain the reconstruction coefficient. Accordingly, we reconstructed the entire wavefront. Figure 9 shows the wavefront reconstruction results from different distances in the water tank with turbulence. It is more intuitive to show underwater turbulence at different distances.

In general, the existing detection methods are ineffective for detecting underwater turbulence, because of the scattering problem. As shown in Figure 9, we are able to detect turbulence changes at different distances using the proposed gated wavefront sensing. The reconstructed wavefronts were calculated in radians where the coefficients were multiplied by 2π/λ. λ is the wavelength. The vertical axis is the phase in radians, and the *xy* plane, is the space where the phase is measured. At the distance of 4 m from our sensing system (where turbulence originated from the water flow in), the wavefront reconstruction model is relatively volatile. At distances further away from the water inlet, the turbulence effect becomes weaker, and the reconstructed wavefront image gradually flattens out, as shown in Figure 9. This verified that the underwater turbulence can be detected by the proposed gated wavefront sensing system.

**Figure 10:**
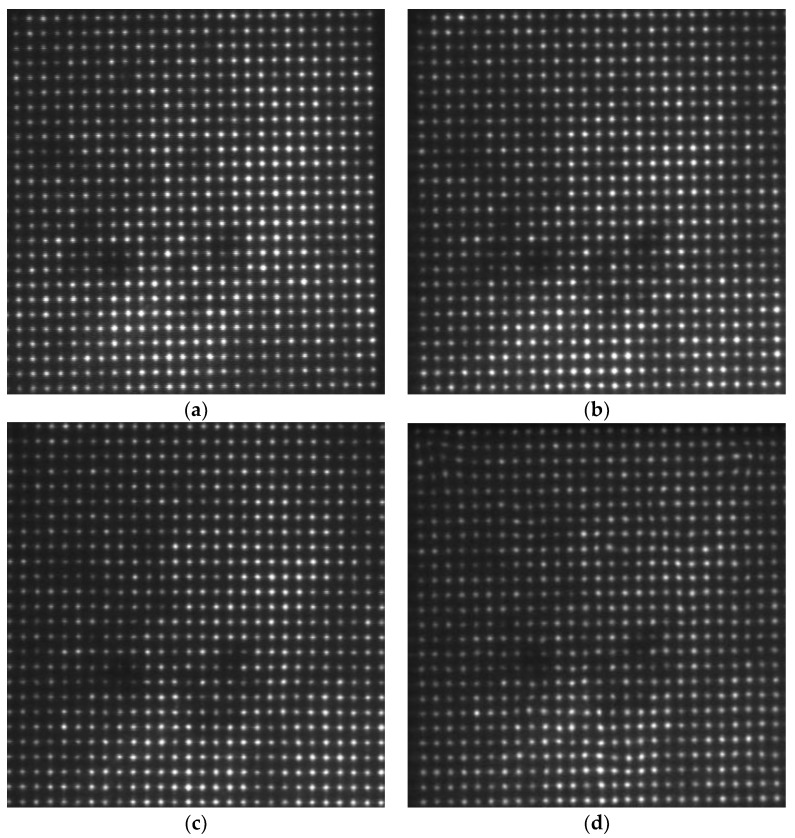
Focal spot images acquired from the same distance i.e., 4 m with different water flow rates: (**a**) 10 L/min; (**b**) 20 L/min; (**c**) 30 L/min; (**d**) 40 L/min.

**Figure 11:**
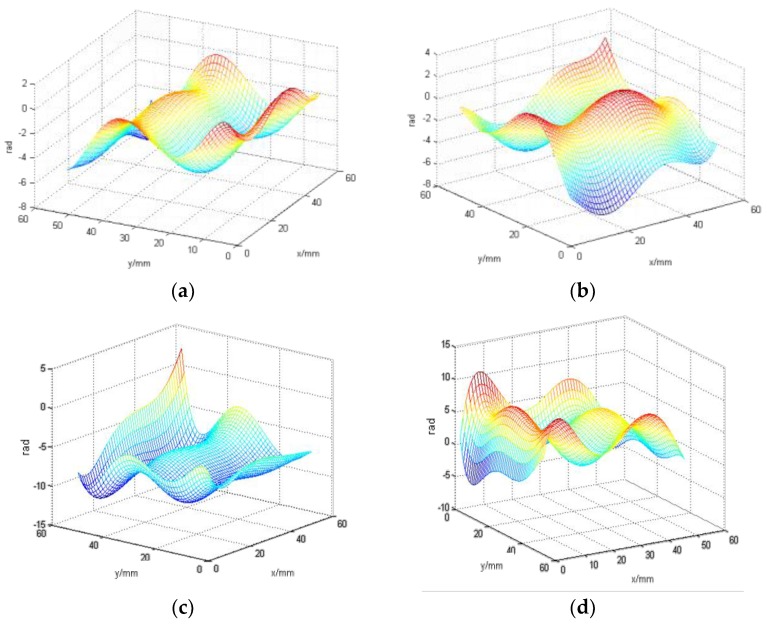
The results of wavefront reconstruction at the same distance with different water flow rates: (**a**) 10 L/min; (**b**) 20 L/min; (**c**) 30 L/min; and (**d**) 40 L/min.

In our next set of experiments, we set the distance at 4 m (where the flow rate can be controlled to create a turbulence condition) and varied the velocity of water flow. Figure 10 shows the focal spot images acquired from the same distance i.e., 4 m, with different water flow rates. Figure 11 shows the wavefront reconstruction results for 10 L/min, 20 L/min, 30 L/min, and 40 L/min, respectively.

Using the reference wavefront obtained from a flow rate of 0 L/min, the wavefronts above in Figure 11 were obtained. As the turbulence generated is spontaneous and impromptu, repeated measurements would not yield similar wavefronts. Therefore, these wavefronts were characterized based on the relative difference between the above spot images and the reference 0 L/min spot image. It can be seen that the peak-to-valley ratio of the reconstructed wavefronts relative to the reference wavefront increased as the flow rate increased. Therefore, there was indeed distortion in the wavefronts due to the induced turbulence from the turbulent flow of water at 4 m. 

As shown in Figure 11, our method is able to detect turbulence changes at different flow rates of water using the proposed gated wavefront sensing system. At the distance of 4 m from our sensing system (where turbulence originated from the water flow in), the higher the water flow rates, the more volatile the wavefront reconstruction model will be. This proves the validity of the proposed gated wavefront sensing system for detecting underwater turbulence.

## 4. Conclusions

In this paper, we proposed a novel method to detect underwater turbulence using a gated wavefront sensing system. The proposed method incorporated wavefront sensing and the range gated approach for effective underwater turbulence detection. Based on the operating principle of this technique, the laser emission and camera gating are simultaneously controlled to only capture the reflection from a known distance. The turbulence condition can be detected from the resulting wavefront reconstruction accordingly. An experimental platform was setup to validate the proposed gated wavefront sensing system. Our experimental results prove that the proposed method can detect underwater turbulence conditions at different distances, and for different levels of turbulence. The aggregated wavefront distortion is successfully detected at increasing depths of the turbulent media. Due to the effectiveness of the proposed method, it has good potential, which will significantly benefit applications in underwater imaging, laser communication, oceanic exploration, etc.

## References

[1] Wang J., Li B., Chen L., Li L. (2017). A novel detection method for underwater moving targets by measuring their ELF emissions with inductive sensors. Sensors.

[2] Lueck R.G., Wolk F., Yamazaki H. (2002). Oceanic velocity microstructure measurements in the 20th century. J. Oceanogr..

[3] Ma W., Wang Y., Xu T. (2017). Design and Sea Trials of the Underwater Glider for Micro-structure Turbulence Measurement. J. Mech. Eng..

[4] Zhang L., Sun C.D., Jun H. (2010). Study on underwater imaging system based on imaging adaptive optics. Appl. Opt..

[5] Sun C.D., Chen L.Y., Gao L.M. (2000). Water optical properties and their effect on underwater imaging. Appl. Opt..

[6] Chen Z., Zhang Z., Dai F., Bu Y., Wang H. (2017). Monocular vision-based underwater object detection. Sensors.

[7] Levine E.R., Lueck R.G. (1999). Turbulence measurement from an autonomous underwater vehicle. J. Atmos. Ocean. Technol..

[8] Macoun P., Lueck R. (2010). Modeling the spatial response of the airfoil shear probe using different sized probes. J. Atmos. Ocean. Technol..

[9] Ilker F., Peterson A.K., Ullgren J.E. (2013). Microstructure measurements from an underwater glider in the turbulent faroe bank channel overflow. J. Atmos. Ocean. Technol..

[10] Fang L., Wang Y., Wang S. (2014). Development of the hybrid underwater glider petreI-II. Sea Technol..

[11] Lueck R.G. (1997). Turbulence Measurement with a Moored Instrument. J. Atmos. Ocean. Technol..

[12] Fan S., Woolsey C. (2013). Elements of underwater glider performance and stability. Mar. Technol. Soc. J..

[13] Levine B.M., Martinsen E.A., Wirth A., Jankevics A., Toledoquinones M., Landers F., Bruno T.L. (1998). Horizontal line-of-sight turbulence over near-ground paths and implications for adaptive optics corrections in laser communications. Appl. Opt..

[14] Azucena O., Crest J., Kotadia S., Kubby J. (2011). Adaptive optics wide-field microscopy using direct wavefront sensing. Opt. Lett..

[15] Lombardo M., Serrao S., Devaney N., Parravano M., Lombardo G. (2013). Adaptive Optics Technology for High-Resolution Retinal Imaging. Sensors.

[16] Sulai Y.N., Dubra A. (2014). Non-common path aberration correction in an adaptive optics scanning ophthalmoscope. Biomed. Opt. Express.

[17] Solano-Altamirano J.M., Vázquez-Otero A., Khikhlukha D., Dormido R., Duro N. (2017). Using Spherical-Harmonics expansions for optics surface reconstruction from gradients. Sensors.

[18] Forest C.R., Canizares C.R., Neal D.R., Mcguirk M., Schattenburg M.L. (2004). Metrology of thin transparent optics using Shack-Hartmann wavefront sensing. Opt. Eng..

[19] Platt B.C., Shack R. (2001). History and principles of Shack-Hartmann wavefront sensing. J. Refract. Surg..

[20] Lane R.G., Tallon M. (1992). Wave-front reconstruction using a Shack–Hartmann sensor. Appl. Opt..

[21] Thibos L.N., Hong X. (1999). Clinical applications of the Shack-Hartmann aberrometer. Optom. Vis. Sci..

[22] Restaino S.R., Hou W., Kanaev A. (2015). Wavefront Sensing and Analysis for Underwater Laser Propagation.

[23] Perreault J.A., Bifano T.G., Levine B.M. (1999). Adaptive Optic Correction Using Silicon Based Deformable Mirrors.

[24] Fried D.L. (2001). Adaptive Optics Development, a 30-Year Personal Perspective.

[25] Zhang Y., Liu Y., Wang S. (2009). Digital mirror device application in reduction of wave-front phase errors. Sensors.

[26] Dong B., Li Y., Han X.-L., Hu B. (2016). Dynamic aberration correction for conformal window of High-Speed aircraft using optimized Model-Based wavefront sensorless adaptive optics. Sensors.

[27] Restaino S.R., Hou W., Kanaev A., Matt S., Font C. (2014). Adaptive Optics Correction of a Laser Beam Propagating Underwater.

[28] Goncharovm A.V., Devaney N., Dainty C. (2007). Atmospheric dispersion compensation for extremely large telescopes. Opt. Express.

[29] Wu C., Ko J., Davis C.C. (2015). Determining the Phase and Amplitude Distortion of a Wavefront using a Plenoptic Sensor. J. Opt. Soc. Am. A-Opt. Image Sci. Vis..

[30] Amann M., Bosch T., Lescure M., Myllyla R., Rioux M. (2001). Laser ranging: A critical review of usual techniques for distance measurement. Opt. Eng..

[31] Yan L., Chen I.-M., Guo Z., Lang Y., Li Y.A. (2011). three degree of-freedom optical orientation measurement method for spherical actuator applications. IEEE Trans. Autom. Sci. Eng..

[32] Ning Y., Li L., Qiu S. Underwater range-gated laser imaging system design with video enhancement processing. Proceedings of the IEEE International Symposium on Instrumentation and Measurement, Sensor Network and Automation.

[33] Wang X.W., Zhou Y., Fan S.T., He J., Liu Y.L. (2010). Range-Gated laser stroboscopic imaging for night remote surveillance. Chin. Phys. Lett..

[34] Sun L., Wang X., Liu X. (2016). Lower-upper-threshold correlation for underwater range-gated imaging self-adaptive enhancement. Appl. Opt..

[35] Matwyschuk A. (2016). Direct method of three-dimensional imaging using the multiple-wavelength range-gated active imaging principle. Appl. Opt..

[36] Wang S.Z., Sun F., Zhang X. (2008). Development of laser illuminating range-gated imaging technique. Infrared Laser Eng..

[37] Wang X., Liu X., Ren P., Sun L., Fan S., Lei P., Zhou Y. (2016). Underwater Three-Dimensional Range-Gated Laser Imaging Based on Triangular-Range-Intensity Profile Spatial-Correlation Method.

[38] Sansoni G., Trebeschi M., Docchio F. (2009). State-of-the-art and applications of 3D imaging sensors in industry, cultural heritage, medicine, and criminal investigation. Sensors.

[39] Massot-Campos M., Oliver-Codina G. (2015). Optical sensors and methods for underwater 3D reconstruction. Sensors.

[40] Hou W., Jarosz E., Woods S. (2013). Impacts of underwater turbulence on acoustical and optical signals and their linkage. Opt. Express.

[41] Kanaev A., Gladysz S., Barros R.A. (2016). Measurements of Optical Underwater Turbulence under Controlled Conditions.

[42] Matt S., Hou W., Goode W. (2017). Introducing SiTTE: A controlled laboratory setting to study the impact of turbulent fluctuations on light propagation in the underwater environment. Opt. Express.

[43] McLean E.A., Burris H.R., Strand M.P. (1995). Short-pulse range-gated optical imaging in turbid water. Appl. Opt..

[44] Wang J., Podoleanu A.G. (2011). Time-Domain Coherence-Gated Shack-Hartmann Wavefront Sensor.

[45] Wang X., Tan C.S., Menoni C. (2015). Multi-Layer Surface Profiling Using Gated Wavefront Sensing.

[46] Tan C.S., Wang X., Ng Y.H., Lim W.K., Chai T.Y. (2013). Method for Distortion Correction of Multi-layered Surface Reconstruction using Time-gated Wavefront Sensing Approach. J. Eur. Opt. Soc. Rapid Public.

[47] Rostami M., Michailovich O., Wang Z. (2012). Image Deblurring Using Derivative Compressed Sensing for Optical Imaging Application. IEEE Trans. Image Process..

